# Skeletal muscle PLIN3 and PLIN5 are serine phosphorylated at rest and following lipolysis during adrenergic or contractile stimulation

**DOI:** 10.1002/phy2.84

**Published:** 2013-09-17

**Authors:** Rebecca E K MacPherson, Rene Vandenboom, Brian D Roy, Sandra J Peters

**Affiliations:** Department of Kinesiology, Centre for Bone and Muscle Health, Brock UniversitySt Catharines, Ontario, L2S 3A1, Canada

**Keywords:** ADRP, exercise, lipolysis, OXPAT, TIP47

## Abstract

In adipose tissue, access of adipose triglyceride and hormone-sensitive lipases (ATGL and HSL) to the lipid droplet depends on PLIN1 phosphorylation, however, PLIN1 is not expressed in skeletal muscle and the phosphorylation of the expressed PLINs has yet to be investigated. Further, direct interactions between skeletal muscle PLINs and HSL are unknown. We investigated the isolated and combined effects of epinephrine and contraction on PLIN-to-lipase interactions as well as phosphorylation. Isolated rat solei were assigned to one of four 30 min in vitro conditions (25°C): (1) rest; (2) intermittent tetanic stimulation (60 Hz for 150 msec; train rate 20/min); (3) 5 nmol/L epinephrine; (4) intermittent tetanic stimulation and 5 nmol/L epinephrine. Immunoprecipitation of serine phosphorylated proteins followed by Western blotting for PLIN2, PLIN3, PLIN5, revealed that only PLIN2 is not phosphorylated under any of the experimental conditions. This is the first study to show that in whole rat skeletal muscle PLIN3 and PLIN5 are serine phosphorylated. The degree of serine phosphorylation remained unchanged following adrenergic and/or contractile stimulation. Oil red O staining of muscle sections for lipid content shows a significant decrease following each condition, confirming lipolysis occurred (*P* < 0.05). PLIN2, 3, and 5 all interact with HSL and ATGL, but these interactions were unchanged following treatments. Our results show that in skeletal muscle, PLIN2 is not serine phosphorylated at rest or with lipolytic stimulation and that while PLIN3, PLIN5 are serine phosphorylated at rest, the degree of phosphorylation does not change with lipolytic stimulation.

## Introduction

Intramuscular triglycerides (IMTGs) represent an important energy source that can be mobilized during exercise through a combination of external hormonal (increased epinephrine) and internal metabolic signals (intracellular Ca^2+^ and metabolite concentrations). However, the exact mechanisms regulating IMTG breakdown during exercise are poorly understood. IMTGs are stored in metabolically active organelles known as lipid droplets that are encased by a phospholipid monolayer coated with a variety of proteins (Londos et al. 1999; Bartz et al. 2007). Evidence indicates that the regulation of skeletal muscle lipolysis is mediated by protein–protein interactions occurring on the lipid droplet surface (Prats et al. 2006; Macpherson et al. 2013). Specifically, a family of lipid droplet proteins, known as PLIN proteins, have emerged as likely candidates in mediating the hydrolysis of IMTGs (Brasaemle et al. 2004; Macpherson et al. 2013). To date, work investigating the role(s) of PLINs has focused on adipose tissue, however, recent investigations in skeletal muscle support a role for PLIN proteins in the regulation of IMTG degradation (Macpherson et al. 2012, 2013; Shaw et al. 2012; Shepherd et al. 2012, 2013).

The PLIN family is composed of five members (PLIN1 through PLIN5) (Miura et al. 2002; Kimmel et al. 2010), each with a unique tissue distribution and potentially a unique role in cellular lipid metabolism (Wolins et al. 2006; Hsieh et al. 2012). PLIN1 is the only member of this family for which a specific role in regulating lipolysis has been determined, however, it is only expressed in adipose tissue. More specifically, in a basal state PLIN1 limits the activity of the rate-limiting lipase, adipose triglyceride (ATGL), by directly binding to its coactivator, CGI-58 (Brasaemle et al. 2000; Souza et al. 2002; Tansey et al. 2003; Miyoshi et al. 2006). Under lipolytic stimulation, initiated by catecholamines, it is believed that the protein kinase A (PKA)-dependent serine phosphorylation of PLIN1 initiates lipolysis by releasing CGI-58 and allowing it to bind to and activate ATGL (Egan et al. 1990; Granneman et al. 2007, 2009; Granneman and Moore 2008; Bezaire and Langin 2009). Further, phosphorylation of PLIN1 is required for hormone-sensitive lipases (HSL) recruitment to the lipid droplet through binding to PLIN1 (Wang et al. 2009). Skeletal muscle does not express PLIN1 and, thus far, similar roles for skeletal muscle PLIN proteins have yet to be determined.

It has been suggested that PLIN2, PLIN3, and PLIN5 play a large role in regulating lipolysis in skeletal muscle (Macpherson et al. 2012, 2013; Peters et al. 2012; Shaw et al. 2012; Shepherd et al. 2012, 2013). PLIN2 is the predominant lipid droplet associated protein in skeletal muscle (Phillips et al. 2005) and PLIN5 is unique in that it is highly expressed in oxidative tissues (Wolins et al. 2006). Although knowledge of PLIN3 in skeletal muscle is scarce, recent work from our laboratory showed that ATGL interacts with PLIN2, PLIN3, and PLIN5 in isolated rat soleus muscle (Macpherson et al. 2013). These protein interactions suggest that PLIN2, PLIN3, and PLIN5 may have a role in the regulation of ATGL activity and therefore the initiation of skeletal muscle lipolysis. In adipose tissue, the reversible phosphorylation of PLIN1 is necessary for lipolytic activation (Su et al. 2003; Sztalryd et al. 2003; Marcinkiewicz et al. 2006; Miyoshi et al. 2006), however, the phosphorylation state of the remaining PLINs has yet to be investigated in skeletal muscle. A phosphorylation site has been identified on PLIN2 (serine 291) (Bartz et al. 2007), PLIN3 (serine 245) (Hickenbottom et al. 2004), and some evidence indicates that PLIN5 is a substrate for PKA phosphorylation (Wang et al. 2011). It is possible that the phosphorylation of PLIN2, PLIN3, or PLIN5 may be required to optimally position ATGL, CGI-58, and/or HSL for activation of lipolysis in skeletal muscle.

Exercise leads to the activation of several skeletal muscle kinases, all of which may play into regulating the rate of lipolysis. Increased circulating epinephrine concentrations leads to the activation of PKA, while at the same time contraction increases intramuscular calcium levels and CaMK and ERK activation. The use of ATP during contraction also leads to increased levels of AMP, which activates AMPK. It is known that epinephrine and contraction activate skeletal muscle hormone-sensitive lipase (HSL) with additive effects, thus indicating that epinephrine and contraction activate HSL through different signaling mechanisms (Spriet et al. 1986; Hopp and Palmer 1990; Dyck and Bonen 1998; Donsmark et al. 2003, 2005; Langfort et al. 2003; Watt et al. 2003). Therefore, the purpose of this study was to examine the isolated and additive effects of epinephrine and contraction on skeletal muscle PLIN protein to ATGL and HSL interactions as well as phosphorylation status. A major objective of this study was to determine if PLIN2, PLIN3, and/or PLIN5 are phosphorylated and whether this changes during lipolysis. If so, this study aimed to separate adrenergic and/or contractile stimulation. A second objective of this study was to investigate the role of PLIN2, PLIN3, and PLIN5 in governing the accessibility of ATGL and HSL via direct protein–protein interactions during these perturbations. We hypothesized that skeletal muscle PLIN-to-lipase interactions are governed by PLIN phosphorylation status.

## Methods

### Animals

A total of 48 male Long-Evans rats (4- to 6-week-old) were used in this study. Animals were housed in groups within the Brock University Animal Facility, where they were maintained on a 12:12-h light–dark cycle at 22°C. The rats were fed a standard rodent diet with ad libitum access to food and water. All experimental procedures and protocols were approved by the Brock University Animal Care and Utilization Committee and conformed to all Canadian Council on Animal Care guidelines.

### Muscle preparation

Animals were anesthetized via intraperitoneal injection of pentobarbital sodium (6 mg/100 g body weight). The left and right solei were removed and placed in organ baths, where they were assigned to one of four experimental conditions: (1) rest; (2) electrically stimulated contraction (Dyck and Bonen 1998; Macpherson et al. 2012); (3) 5 nmol/L epinephrine (Peters et al. 1998); and (4) epinephrine and electrical stimulation. To briefly summarize the preparation, each soleus muscle was dissected tendon-to-tendon, sutures tied in situ, the muscle was then removed and immediately placed in an organ bath (Radnoti Glass Technology, Monrovia, CA), which contained 7–8 mL of fully oxygenated liquid Sigma medium 199 (M 4530; Sigma-Aldrich, Oakville, Canada) and suspended at a resting tension of 1 g. The incubation medium was continuously gassed with 95% O_2,_ 5% CO_2_, and temperature was maintained at 25°C (Antolic et al. 2007). All muscles were allowed to equilibrate at rest for 30 min. This isolated muscle preparation allows for the examination of the isolated effects of epinephrine, contraction, as well as epinephrine and contraction together on muscle lipid metabolism in isolation, absent from other systemic perturbations.

### Perturbation

#### Epinephrine incubation

Soleus muscles were stimulated to contract for 30 min as previously reported (Dyck and Bonen 1998; Macpherson et al. 2012, 2013). This concentration of epinephrine has previously proven to maximally promote triglyceride breakdown in isolated soleus muscles (Peters et al. 1998).

#### Stimulated contraction

Soleus muscles were stimulated to contract for 30 min as previously reported by our lab and others (Dyck and Bonen 1998; Macpherson et al. 2012, 2013). Initially, optimal stimulus voltage was determined by assessing force responses (Grass Telefactor force transducer, West Warwick, RI) to single electrical pulses (Grass Model FT03 with P11T amplifier). Stimulus intensity was increased from 10 V in 10-V increments, until a plateau in twitch force was reached, after which stimulus voltage was increased to ∼1.25 of this level. During the 30-min stimulus protocol, muscles received repeated volleys of brief (150 msec) but high-frequency (60 Hz) trains at a train rate of 20 tetani/min while muscles were suspended at 1 g of resting tension. This protocol was previously proven to elicit maximal rates of triglyceride pool turnover and rates of IMTG oxidation without the development of fatigue (Dyck and Bonen 1998; Macpherson et al. 2012). Throughout this period, muscle force was recorded using Grass Polyview Data Acquisition and Analysis System (West Warwick) and analyzed using the Polyview Reviewer (Grass Polyview Data Acquisition and Analysis System; Astro-Med, West Warwick).

#### Epinephrine and electrically stimulated contraction

Soleus muscles were incubated with 5.0 nmol/L epinephrine as well as electrically stimulated to contract, described above, for 30 min.

### Sample preparation

Following the incubations, solei were removed from the baths and cut into two pieces. One piece was snap frozen in liquid nitrogen for Western blotting analysis and the other piece was mounted for histochemical analysis (see below). Soleus muscles were homogenized in Griffin lysis buffer (150 mmol/L NaCl, 50 mmol/L Tris HCl, 1 mmol/L ethylene glycol tetraacetic acid [EGTA]) using a 1:25 dilution of muscle to buffer with added protease (11836170001, Roach, QC), and phosphatase inhibitor tablets (04906845001, Roach, QC). For measures of protein phosphorylation a kinase inhibitor (7,8-dihydroxycoumarin, 1001251297, Sigma-Aldrich) was added to the homogenization buffer. Protein concentration of the total homogenates was determined using a Bradford Assay (Bio-Rad Protein Assay Dye Reagent Concentrate; #500-0006; Bio-Rad, Mississauga, ON, Canada).

### Protein interactions (coimmunoprecipitation)

Sample homogenates were immunoprecipitated with 5 μL of the appropriate primary antibody (ATGL or HSL) and then immunoblotted for the corresponding protein (PLIN2; PLIN3; PLIN5; CGI-58). Specifically, 500–1000 μg of protein from each sample were incubated for 2 h with the antibody at 4°C. Pilot work was done in order to determine the appropriate amount of whole homogenate to incubate with the antibody in order to fully isolate the protein of interest. Following this 20 μL of Protein G Agarose beads (sc-2001; Santa Cruz Biotechnology, Inc., Dallas, TX) were added to each sample for overnight incubation at 4°C. The pellet of each sample was collected by centrifugation at 130 rpm for 5–10 sec. Pellets were washed three times in phosphate buffered saline (PBS) and resuspended in 40 μL of 2× sample buffer. To test for antibody interference in the samples a blank sample containing only the precipitating antibody and lysis buffer were prepared in exactly the same manner as the experimental samples. For interactions where antibody interference occurred, a secondary antibody that only detects native (i.e., not denatured) antibodies was used (Clean Blot IP Detection Reagent; Thermo Scientific, Rockford, IL). All samples were boiled and separated using 8 or 10% SDS-PAGE (sodiumdodecyl sulphate polyacrylamide gel electrophoresis)–polyacrylamide gel electrophoresis.

### Protein phosphorylation (immunoprecipitation of PSer proteins)

To ensure immunoprecipitation of all serine phosphorylated proteins sample homogenates were immunoprecipitated with 40 μL of antiphosphoserine antibody (Millipore AB1603, Billerica, MA) and then immunoblotted for the corresponding protein (ATGL; PLIN2; PLIN3; PLIN5). Specifically, 500 μg of protein from each sample were used in order to isolate PSer proteins. In order to avoid antibody interference as well as save sample these samples were prepared using a Pierce^**®**^ immunoprecipitation kit (#26149; Thermo Scientific). A phosphoserine antibody was used based on previous work indicating that PLIN2, PLIN3, and ATGL contain serine phosphorylation sites (Hickenbottom et al. 2004; Bartz et al. 2007; Mason et al. 2012), and that PLIN5 may be substrate for PKA (Wang et al. 2011).

### Western blotting

SDS-polyacrylamide gel electrophoresis (8 or 10% separating; 4% stacking) was used to separate proteins (ATGL, CGI-58, PLIN2, PLIN3, and PLIN5) at 120 V for 1.5 h, and proteins were electroblotted onto polyvinylidene difluoride membranes (Amersham Biosciences, Piscataway, NJ) for 1 h at 100 V followed by blocking in 2 or 5% fat-free milk in Tris buffered saline with Tween 20 (TBST) or 5% bovine serum albumin (BSA) in TBST. Primary antibodies for coprecipitated proteins were diluted 1:1000 in 2 or 3% fat-free milk or 1 or 5% BSA in TBST and incubated overnight at 4°C. Secondary antibodies were diluted 1:10,000–20,000 in 2 or 3% milk or 1 or 5% BSA and incubated for 1 h. Blots of specific proteins were visualized with enhanced chemiluminesence (Amersham Biosciences). The densities of the individual bands were integrated using Image J software (http://rsbweb.nih.gov/ij/). Each blot had loaded whole soleus homogenate as a positive control for the coprecipitated protein. Blots were normalized to total protein loaded determined by Ponceau S staining (M530; Sigma-Aldrich) and results are reported as the ratio of the density of the target protein to the density of the loaded protein in arbitrary units (Romero-Calvo et al. 2010; Macpherson et al. 2013).

### Antibodies

The following antibodies were used and have been used previously: antiphosphoserine antibody (Millipore AB1603), PLIN2 (52 kDa) mouse monoclonal antibody (Cat. No. 610102; Progen Biotechnik, Heidelberg, Germany) (Peters et al. 2012; Macpherson et al. 2013), PLIN3 (47 kDa) (Cat. No. 3883; ProSci Inc., Poway, CA) (Peters et al. 2012; Macpherson et al. 2013), PLIN5 (52 kDa) guinea pig polyclonal antibody (Cat. Nos. GP34 and GP31; Progen Biotechnik) (Minnaard et al. 2009; Bosma et al. 2012b; Peters et al. 2012; Macpherson et al. 2013), ATGL (54 kDa) rabbit monoclonal antibody (#2439; Cell Signalling Technology, Danvers, MA) (Alsted et al. 2009; Macpherson et al. 2013), CGI-58 (42 kDa) rabbit polyclonal antibody (Novus Biologicals, NB110-41576, Oakville, ON) (Alsted et al. 2009; Timmers 2011; Macpherson et al. 2013), and HSL (4107S; Cell Signalling Technology).

### Histochemical analysis

The muscle section used for histochemical analysis was oriented for cross-sections and mounted, in embedding medium (Cryomatrix, Pittsburgh, PA), on a piece of cork, which was plunged into 2-methylbutane cooled in liquid nitrogen. Following rapid freezing, the samples were stored at −80°C until sectioning. Sectioning was completed with a cryotome (ThermoShandon, Runcorn, Cheshire, U.K.) optimally set at −20°C. Sections were (10 μm thick) thaw mounted onto slides and stored at −80°C until histochemical staining. To permit the examination of lipid droplets, oil red O (ORO; O0625; Sigma-Aldrich, St. Louis, MO) (Koopman et al. 2001) was utilized (Koopman et al. 2001; van Loon et al. 2003, 2004; Stellingwerff et al. 2007). Briefly, cryosections were fixed in 3.7% formaldehyde for 1 h. Slides were then rinsed three times in deionized water for 30 sec, and then immersed in the working solution of ORO for 30 min. Slides were rinsed three times in deionized water and cover slips were mounted with a prolonging agent (no. P36930; Prolong Gold anti-fade reagent; Invitrogen, Burlington, ON, Canada).

### Image capturing and analyses

All sections were examined using a Nikon Eclipse 80i fluorescence microscope (Nikon Eclipse 80i; Chiyoda-ku, Tokyo, Japan). Digital images of the slides were captured with a digital camera (Retiga 1300, QImaging, Burnaby, BC, Canada) attached to the microscope. To visualize the ORO stain a TRITC (510–560 nm) excitation filter was used. Digitally captured images (×40 magnification), four fields of view/muscle cross section (23.1 ± 0.6 fibers/field of view), were processed and analyzed using imaging software (NIS-Elements AR 3.00; Nikon Instruments, Melville, NY). An intensity threshold representing minimal values corresponding to lipid droplets was set manually and applied uniformly in all images. The lipid droplet fluorescent signals were quantified for each muscle fiber, resulting in a total of 92.3 ± 2.6 fibers analyzed for each muscle cross section. Fiber area, as well as number and area of objects emitting a fluorescent signal, were recorded. Muscle fiber lipid droplet content was expressed as the fraction of the measured area that was stained per fiber (van Loon et al. 2004; Macpherson et al. 2012). All measures were manually outlined and traced by investigators for each individual myocyte. The immunofluorescence method described here covers numerous fibers per muscle cross section and, therefore, gives a good representation of the entire muscle. To test the reliability of the method in our hands, both intraobserver and interobserver reliability were evaluated by two investigators. The intraobserver reliability involved the two investigators performing analysis of one image three times, at least 1 week apart. The interobserver reliability involved two independent investigators performing analysis for three separate images. These tests proved to be reliable with a coefficient of variation <5% for both intraobserver and interobserver reliability.

### Statistics

Comparisons of mean lipid content, protein interactions, and phosphorylation between groups were performed using a one-way analyses of variance (ANOVA) (incubation condition). Tukey post hoc tests were performed when significance was detected. Statistical significance was set at *P* < 0.05. All data are expressed as means ± SEM.

## Results

### Lipid droplet content

**Figure 1:**
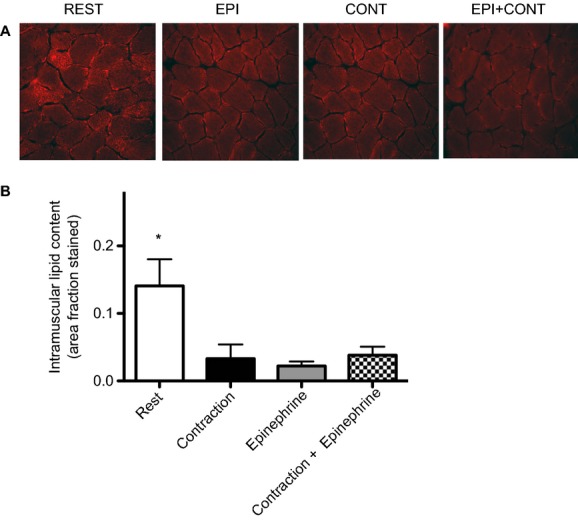
Digitally captured images of one single field of view (×40 magnification) taken from a soleus muscle cross section. (A) Oil red O (ORO) staining. EPI, epinephrine stimulate 5 nmol/L; CONT, electrically stimulated contractions for 30 min; EPI + CONT, combination of 5 nmol/L epinephrine and electrically stimulated contractions for 30 min. (B) Lipid content (expressed as area fraction stained) in rested and stimulated rat soleus muscle. Values are expressed as means ± SE. *Significantly different from experimental conditions (*P* < 0.05) (*n* = 9 for EPI and CONT; *n* = 10 for REST and EPI + CONT).

The average muscle fiber area was 2604 ± 532 μm^2^. Figure 1 shows representative images of rat skeletal muscle cross sections viewed with an immunofluorescence microscope following incubation with ORO. Fiber lipid droplet content (area lipid stained) decreased to ∼30% of resting values in all groups (*P* = 0.001; Fig. 1).

### Protein interactions

**Figure 2:**
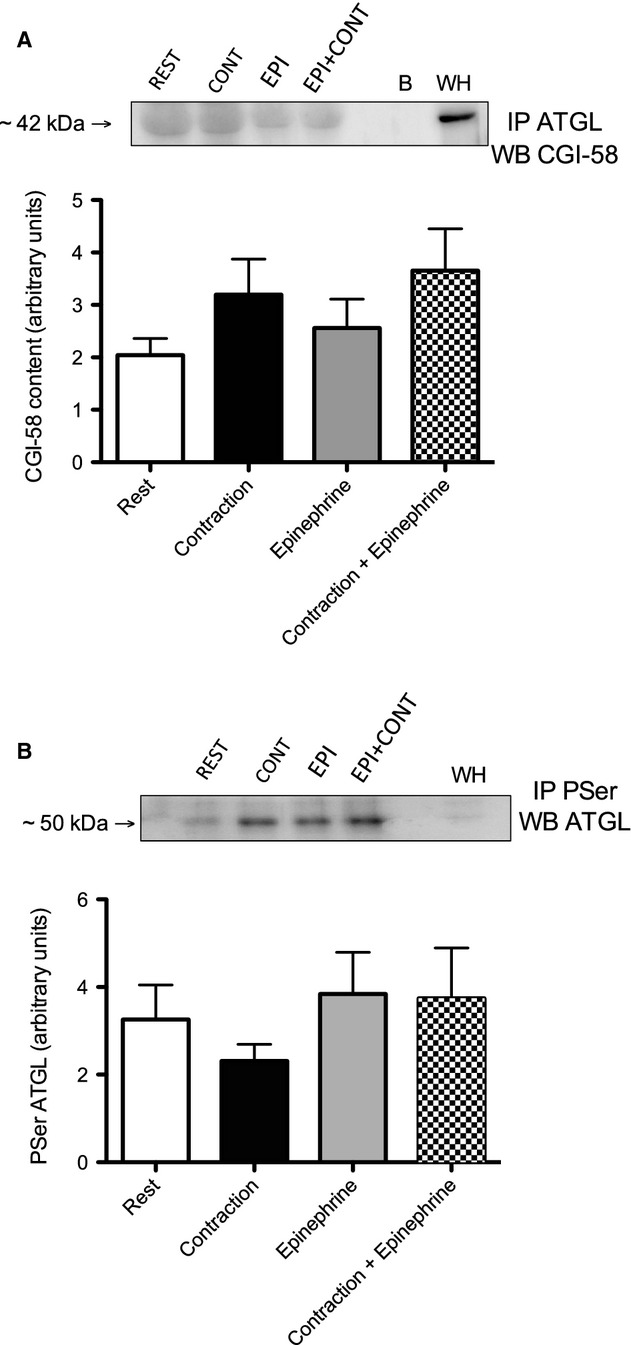
(A) ATGL-CGI-58 interaction: CGI-58 protein content (arbitrary units) in ATGL immunoprecipitated samples at rest and following stimulation with representative Western blot (EPI, epinephrine stimulate 5 nmol/L; CONT, electrically stimulated contractions for 30 min; EPI + CONT, combination of 5 nmol/L epinephrine and electrically stimulated contractions for 30 min; B negative control containing just the IP antibody; WH, whole homogenate) (*n* = 12 for REST and CONT + EPI; *n* = 11 for CONT and EPI). (B) ATGL serine phosphorylation: ATGL protein content (arbitrary units) in phosphoserine immunoprecipitated samples at rest and following stimulation with representative Western blots (*n* = 8).

ATGL coimmunoprecipitated with CGI-58 at rest and following each perturbation. Contraction, epinephrine, and the combination appeared to increase this interaction ∼50%, 25%, and 80%, respectively, although this was not statistically significant (*P* = 0.25; Fig. 2).

**Figure 3:**
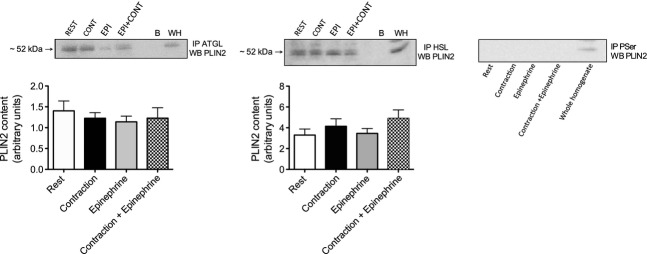
ATGL/HSL-PLIN2 protein interactions and PLIN2 serine phosphorylation at rest and following stimulation shown as PLIN protein content (arbitrary units) in immunoprecipitated samples with representative Western blots (EPI, epinephrine stimulate 5 nmol/L; CONT, electrically stimulated contractions for 30 min; EPI + CONT, combination of 5 nmol/L epinephrine and electrically stimulated contractions for 30 min; B negative control containing just the IP antibody; WH, whole homogenate) (*n* = 10). PLIN2, Western blotting for PLIN2 in phosphoserine immunoprecipitated samples resulted in no detectable signal under any condition.

**Figure 4:**
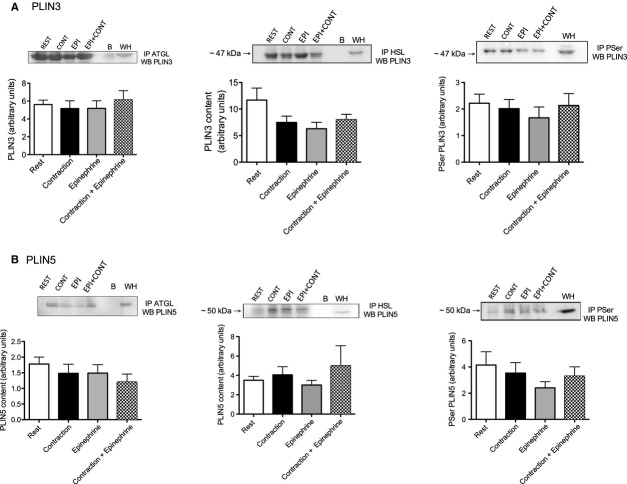
ATGL/HSL-PLIN protein interactions and PLIN serine phosphorylation at rest and following stimulation shown as PLIN protein content (arbitrary units) in immunoprecipitated samples with representative Western blots (EPI, epinephrine stimulate 5 nmol/L; CONT, electrically stimulated contractions for 30 min; EPI + CONT, combination of 5 nmol/L epinephrine and electrically stimulated contractions for 30 min; B negative control containing just the IP antibody; WH, whole homogenate) (*n* = 10). (A) PLIN3. (B) PLIN5.

PLIN2, 3, and 5 all coimmunoprecipitated with ATGL at rest and following each perturbation. There were no significant differences in PLIN protein content in ATGL immunoprecipitated samples following any of the perturbations (*P* > 0.05) (Figs. 3, 4).

PLIN2, 3, and 5 all coimmunoprecipitated with HSL at rest and following each perturbation. There were no significant differences in PLIN protein content in HSL immunoprecipitated samples following any of the perturbations (*P* > 0.05) (Figs. 3, 4).

### Protein serine phosphorylation

Western blotting for PLIN2 protein content in immunoprecipitated serine phosphorylated proteins resulted in no detectable PLIN2 protein at rest or with any of the perturbations (Fig. 3). Phosphorylation of PLIN3, PLIN5, and ATGL was detectable at rest with no significant difference under any of the perturbations (*P* > 0.05) (Fig. 4).

## Discussion

Skeletal muscle PLIN proteins are believed to play a critical role in regulating IMTG turnover, however, the exact mechanisms regulating lipolysis within skeletal muscle during exercise remain unknown. This study investigated the effects of contraction and epinephrine alone and in combination on the phosphorylation state of PLIN2, PLIN3, PLIN5, and ATGL, as well as the PLIN interactions with ATGL and HSL. This is the first study to show in intact rat skeletal muscle that PLIN2, PLIN3, and PLIN5 interact with HSL and that these interactions are unchanged following either contractile or adrenergic stimulation. Further findings from this work demonstrate that both PLIN3 and PLIN5 are serine phosphorylated while PLIN2 is not serine phosphorylated. Contrary to our hypothesis there was no significant change in PLIN3 or PLIN5 phosphorylation with contraction or epinephrine alone or in combination. These novel findings indicate that, unlike adipose tissue where PLIN1 phosphorylation is required for lipolysis, skeletal muscle PLIN proteins are phosphorylated in a basal state and that overall this total phosphorylation does not change with lipolysis under either adrenergic or contractile stimulation. Further work is necessary to investigate the site specific phosphorylation of these two proteins to determine if there are other sites that may have been affected.

Our experimental conditions were chosen for their ability to elicit large levels of IMTG turnover. The stimulated contraction protocol used in this study has been shown to elicit maximal rates of lipolysis in isolated soleus muscle and we have previously shown that this method leads to a significant decline in IMTGs (Dyck and Bonen 1998; Macpherson et al. 2012). Moreover, the epinephrine concentration used (5 nmol/L) was previously shown to increase IMTG hydrolysis in isolated rat soleus (Peters et al. 1998). In the present study, we found a significant reduction in intramuscular lipids stained by ORO following each experimental condition (Fig. 1; electrically stimulated contraction 68% decrease, epinephrine stimulation 65% decrease, and combination of both 73% decrease), thus confirming that our model elicited high rates of lipolysis in all experimental conditions.

We show that the interactions of PLIN2, PLIN3, and PLIN5 with ATGL and HSL are unchanged following contraction or epinephrine stimulation alone or in combination. PLIN2 was the only skeletal muscle PLIN protein not to be serine phosphorylated under any of the experimental conditions (rest, contraction, epinephrine, or both). This finding is interesting, as PLIN2 has been suggested to function similarly to PLIN1 by increasing triglyceride storage and decreasing triglyceride turnover (Imamura et al. 2002; Fukushima et al. 2005; Chang et al. 2006; Imai et al. 2007; Bell et al. 2008). In skeletal muscle, some evidence indicates that PLIN2 is involved in regulating lipolysis, however, other research points toward an essential role of PLIN2 in IMTG synthesis. Recently, Shepherd et al. (2012) found that in human skeletal muscle PLIN2-associated lipid droplets are preferentially used with an acute bout of exercise, supporting a role for PLIN2 in regulating muscle lipolysis. Interestingly, PLIN2 protein content is higher in cases where there is increased IMTG content, such as in females, type II diabetics, and with endurance training (Minnaard et al. 2009; Peters et al. 2012; Shaw et al. 2012). Therefore, a higher PLIN2 content could simply be a consequence of increased PLIN2 protein stability through increased lipid droplet size and number. Previous work with skeletal muscle contraction has shown a translocation of HSL toward PLIN2-coated lipid droplets (Prats et al. 2006), however, this study did not determine if there was a direct physical interaction between these two proteins. Our work demonstrates an interaction between PLIN2 and ATGL (Macpherson et al. 2013) as well as HSL, however, this interaction remains unchanged following contraction and/or epinephrine stimulation in vitro. Previously, our group found that the interaction between PLIN2 and ATGL is significantly decreased (21%) following electrically stimulated contraction (Macpherson et al. 2013). The present study shows a trend for a decline in this interaction following each of the experimental conditions, however, this did not meet statistical significance likely due to the small sample size and the addition of two groups to the statistical analysis. Future work should investigate the importance of this interaction in skeletal muscle. It is possible that PLIN2 may not be directly involved in stimulated skeletal muscle lipolysis but is more involved with IMTG synthesis and lipid droplet growth. PLIN2 content increases with fatty acid loading of myotubes and with in vivo interventions that lead to an increased muscle lipid content (high fat diet and fasting) (Bosma et al. 2012a). Additionally, overexpression of PLIN2 in vitro and in vivo results in increased intramyocellular lipid content. While knockdown of myotube PLIN2 prevents fatty acid induced IMTG accumulation and lipid droplet storage (Bosma et al. 2012a). Due to the association of PLIN2 with both ATGL and HSL it is possible that PLIN2 is important for regulating basal lipolysis, however, future work may want to investigate a role for PLIN2 in lipid droplet development and its relationship to proteins involved in IMTG synthesis.

In this study, both PLIN3 and PLIN5 were found to be serine phosphorylated and this did not change following either adrenergic or contractile stimulation. Also, both PLIN3 and PLIN5 interacted with ATGL and HSL but this was unchanged following lipolytic stimulation. Therefore, like PLIN2, the roles of PLIN3 and PLIN5 in skeletal muscle lipid dynamics remain unclear. Among the PLIN family, PLIN3 has a high degree of sequence similarity with PLIN2 and also shows a ubiquitous tissue expression (Lu et al. 2001; Wolins et al. 2001), therefore PLIN3 may play a similar role as PLIN2 in lipid droplet dynamics. Association of PLIN3 with lipid droplets increases in cells grown in media supplemented with exogenous fatty acids or glucose and insulin (designed to increase triglyceride synthesis) (Wolins et al. 2001, 2005). Moreover, in PLIN2 null mice, PLIN2 on the LD is replaced with PLIN3 (Sztalryd et al. 2006). This research suggests that PLIN3, like PLIN2, plays a role in lipid droplet formation and triglyceride synthesis and less of a role in stimulated lipolysis. PLIN5 has previously been shown to be essential for ATGL-mediated lipolysis (Granneman et al. 2009, 2011) and was found to be a substrate for PKA in AML12 mouse liver cells, suggesting that PLIN5 phosphorylation may be important for lipolysis (Wang et al. 2011). However, our results show that in skeletal muscle, PKA stimulation through epinephrine does not change the serine phosphorylation state of PLIN5 or the association with either ATGL or HSL. PKA is both a serine and threonine kinase and therefore it is possible that PLIN5 is also phosphorylated on a threonine site. This highlights the importance of determining the specific sites on which PLIN5 may be phosphorylated. Further, we show that contraction-induced kinases did not result in changes in the overall serine phosphorylation state of PLIN5 or change the interactions with lipases. Due to the oxidative tissue expression of PLIN5 (Wolins et al. 2006), as well as its association with mitochondria (Bosma et al. 2012b), it is likely that PLIN5 plays a role in facilitating fatty acid oxidation and not IMTG hydrolysis per se. In support of this theory, Peters et al. (2012) found PLIN5 protein content increased with endurance training and this increase was associated with improved muscle oxidative capacity. Other work has demonstrated that overexpression of PLIN5 in vitro increased fatty acid oxidation (Wolins et al. 2006), providing evidence for a larger role of PLIN5 in enhancing the oxidation of the fatty acids released from the lipid droplets over IMTG synthesis or lipolysis. Overexpression of PLIN5 in skeletal muscle also promotes expression of several genes, regulated by PPARα and PGC1α, involved in fatty acid catabolism and oxidation (Bosma et al. 1831). The recent discovery that PLIN5 is located in direct contact with mitochondria and that PLIN5 overexpression results in more intimate interactions between these two organelles provides more evidence in support of a strong role in oxidation (Bosma et al. 2012b). Our findings that PLIN5 is associated with both ATGL and HSL with no change under any condition indicate that PLIN5 may be involved in regulating basal lipolysis but not stimulated lipolysis. Future work should investigate a role for PLIN5 in fatty acid oxidation and mitochondrial association. The mechanistic function of interactions between PLIN2, PLIN3, and PLIN5 with both ATGL and HSL remains to be investigated. These interactions might be essential for maintaining a stable rate of basal lipolysis or ensuring that the lipases are targeted to the right subcellular location.

ATGL is now known as the rate-limiting lipase in lipolysis and its activity is enhanced in the presence of CGI-58 (Zimmermann et al. 2004; Lass et al. 2006). We have previously shown that 30 min of isolated soleus contraction leads to a significant increase in the interaction between ATGL and CGI-58, consistent with the increased rates of lipolysis observed in our study, as well as being consistent with the activation of lipolysis in adipose tissue (Macpherson et al. 2013). However, in the present study, we found that the increased ATGL and CGI-58 interaction did not reach significance with any of our experimental conditions. It has been suggested that a direct physical interaction between ATGL and CGI-58 is not actually necessary to increase the lipolytic activity of ATGL (Lu et al. 2010). Gruber et al. (2010) demonstrated that CGI-58 is able to bind directly to the phospholipid monolayer of lipid droplets and that without this there is a complete loss of the ability of CGI-58 to activate ATGL, regardless of their interaction. These results may explain why in the present study there was no significant increase in protein–protein interaction between ATGL and CGI-58. It may be more important for CGI-58 to bind to the lipid droplet membrane and somehow allow ATGL to access the triglyceride core (Lu et al. 2010).

Previous studies have identified serine phosphorylation sites on ATGL (serine 406/404 and 428/430) (Zimmermann et al. 2004; Bartz et al. 2007), however, functional roles of phosphorylation and the kinases involved remain unknown. While work in other cell types (HEK293 cells and adipocytes) indicates that AMPK (Ahmadian et al. 2011) and PKA (Pagnon et al. 2012) can phosphorylate ATGL, our results show that ATGL serine phosphorylation remains unchanged in the face of contractile and/or adrenergic stimulation. This finding is in agreement with Mason et al. (2012) who demonstrated that ATGL Ser404 phosphorylation is not increased in human skeletal muscle during moderate-intensity cycling exercise. In addition, that study demonstrated that there was no evidence of a physical interaction between ATGL and AMPK after immunoprecipitation, and pharmacological activation of AMPK did not affect ATGL Ser404 phosphorylation in cultured myotubes (Mason et al. 2012). Further, direct studies in C2C12 myotubes showed no effect of forskolin (PKA activation) on ATGL Ser406 phosphorylation (Mason et al. 2012). From this, Mason et al. concluded that neither AMPK nor PKA phosphorylate skeletal muscle ATGL Ser404 in intact cell systems or whole organisms. Taken together with the results from the present study it seems as though there is a tissue-specific regulation of ATGL, potentially explaining the differences seen in skeletal muscle compared to human embryonic kidney cells (HEK) cells and adipocytes.

### Perspectives and significance

This study examined the serine phosphorylation states of skeletal muscle PLIN proteins (PLIN2, PLIN3, and PLIN5) as well as their interactions with ATGL and HSL at rest and following contraction and epinephrine stimulation in isolation as well as in combination. This is the first study to show that both PLIN3 and PLIN5 are serine phosphorylated under all conditions and that PLIN2 is not. Further this is the first study to determine that PLIN2, PLIN3, and PLIN5 all interact with HSL and that this relationship remains unchanged following stimulation. Together, the above-mentioned data suggest that, unlike in adipose tissue where PLIN1 is unphosphorylated at rest, in skeletal muscle PLIN3 and 5 are serine phosphorylation at rest and this is unchanged with lipolysis. The physiological importance of skeletal muscle PLIN3, PLIN5, and ATGL phosphorylation as well as the PLIN-to-lipase interactions remains unknown. A role for skeletal muscle PLIN phosphorylation in lipid droplet dynamics should be further examined with the development of phosphor-specific antibodies. In comparison to adipose tissue skeletal muscle has a much higher triglyceride turnover (Sacchetti et al. 2004). Therefore, it is possible that the PLIN proteins are important for basal muscle lipolysis rather than epinephrine- or contraction-mediated IMTG breakdown. Future work should examine the roles that skeletal muscle PLINs play on lipogenesis as well as fatty acid oxidation.
